# Potency of Dimethyl Dicarbonate on the Microbial Inhibition Growth Kinetics, and Quality of Passion Fruit (*Passiflora edulis*) Juice during Refrigerated Storage

**DOI:** 10.3390/foods13050719

**Published:** 2024-02-27

**Authors:** Khursheed Ahmad Shiekh, Akaranaj Noieaid, Poke Gadpoca, Supassorn Sermwiwatwong, Saeid Jafari, Isaya Kijpatanasilp, Randy W. Worobo, Kitipong Assatarakul

**Affiliations:** 1 Department of Food Technology, Faculty of Science, Chulalongkorn University, Bangkok 10330, Thailand; khursheedahmad.s@chula.ac.th (K.A.S.); 6132593923@alumini.chula.ac.th (A.N.); 6132568223@alumini.chula.ac.th (P.G.); 6132588823@alumini.chula.ac.th (S.S.); saeid.j@chula.ac.th (S.J.); ykijpat@gmail.com (I.K.); 2 Department of Food Science, College of Agriculture and Life Sciences, Cornell University, Ithaca, NY 14853-5701, USA

**Keywords:** dimethyl dicarbonate, microbial inhibition kinetics, pasteurization, passion fruit juice, quality

## Abstract

This study aimed to investigate the effectiveness of dimethyl dicarbonate (DMDC) at various concentrations (0–250 ppm) in inhibiting the growth of *Escherichia coli* TISTR 117 and spoilage microbes in passion fruit juice (PFJ) and its impact on the physicochemical and antioxidant quality of the juice during refrigerated storage. The highest log reduction in the total viable count, yeast/molds and *E. coli* was attained in PFJ samples with 250 ppm of DMDC (*p* ≤ 0.05) added. Microbial growth inhibition by DMDC followed the first-order kinetic model with a coefficient of determination (R^2^) and inhibition constants (k) ranging from 0.98 to 0.99 and 0.022 to 0.042, respectively. DMDC at 0–250 ppm showed an insignificant effect on pH, °Brix, color (L*, a*, b*), ascorbic acid, total phenolic compound (TPC), total flavonoid content, and antioxidant activity (DPPH, FRAP) (*p* > 0.05). Control (untreated PFJ), DMDC-250 ppm, and pasteurized (15 s at 72 °C) samples were subjected to 27 days of cold storage at 4 °C. A decreasing trend in pH, total soluble solid, ascorbic acid content, DPPH and FRAP values were observed in all the samples during refrigerated storage. However, the DMDC-250 ppm sample showed a better prospect in physicochemical quality changes compared to the pasteurized and untreated control PFJ samples. ΔE values showed marked changes in the control sample than the DMDC-250 ppm and pasteurized samples at 27 days of storage. Additionally, the total viable count and yeast/mold count were augmented during storage, and an estimated shelf-life of the control, DMDC-250 ppm, and pasteurized samples was approximately 3, 24 and 18 days, respectively. In conclusion, DMDC at 250 ppm could ensure microbial safety without affecting the quality attributes of PFJ during 24 days of storage at 4 °C.

## 1. Introduction

Consumer choices are increasing nowadays toward fresh fruits and vegetables that provide bioactive compounds with antioxidant effects to promote a healthy lifestyle [1]. The purple passion fruit (*Passiflora edulis*) is a valuable fruit in Thailand because of its sweet aroma, sour taste due to organic acids and vital nutrients and is served as a refreshing fruit juice [2]. Passion fruit is an abundant source of polyphenols, vitamin C, total carotenoids, flavonoids, tannins and saponins that have potential antioxidant and nutraceutical properties [3]. Catechin and β-carotene are the main phytochemicals constituents in yellow, orange and purple passion fruit cultivars [4]. Furthermore, passion fruit phytochemicals have been subjected to in vivo biological studies to examine their antioxidant, anti-inflammatory, antibacterial, and antifungal properties, which have demonstrated encouraging potential in promoting health and combating various diseases such as pulmonary fibrosis, hypertension, asthma, osteoarthritis, and diabetes [5]. Concerning the storage of passion fruit juice (PFJ), several pathogens and spoilage microbes have been a challenge for the fruit juice industry. *Alicyclobacillus acidoterrestris* and *Neosartorya fischeri* being non-pathogen microbes are responsible for the spoilage of PFJ during storage [6]. The ascospores such as the *Byssochlamys fulva*, *Neosartorya fischeri*, and *Talaromyces* species are heat-resistant fungi that contaminate PFJ and make it unsafe for consumers [7].

Functional components and nutritional quality are mostly affected by chemical and microbial spoilage during the storage of fruit juices. To avoid encountering such quality limitations, traditional pasteurization, non-thermal techniques, and the use of safe additives have been employed to maintain the quality and prolong the shelf-life of fruit juices during extended periods of refrigerated storage [8]. However, conventional pasteurization may affect heat sensitive phytochemical and nutritional constituents, while non-thermal technologies are still not accessible to small-scale juice processing industries because of the higher cost setup [9,10]. In addition, dimethyl dicarbonate (DMDC) potentially eliminates microbial spoilage without affecting the quality attributes and nutritional components of fruit [11]. DMDC is a safe additive and has been employed in the preservation of biochemical quality and extend the shelf-life of mango and passion fruit smoothies [12] and pomegranate juice [13] during cold storage.

DMDC has been added to beverages to act as a potential antimicrobial agent against pathogenic and spoilage microorganisms. The application of DMDC (250 ppm) reduced the number of *E. coli* O157: H7 and *S. aureus* by 5 and 1 log CFU/mL, respectively, at 4 °C for 2 h in mango and passion fruit smoothie [12]. DMDC at 250 ppm inhibited kinetically pathogenic and spoilage microorganisms (*E. coli*, *Saccharomyces cerevisiae* and *Lactobacillus plantarum*) in pomegranate juice during cold storage (4 °C) [13]. The United States Food and Drug Administration (2000) recommended a 250 ppm of DMDC dosage limit in non-carbonated 100% juice beverages [14]. DMDC interacts with histidyl protein residues of enzymes inside the microbial cell by blocking active sites or inducing conformational changes, rendering the malfunctioning of cellular metabolism which leads to cell death [15]. However, there are no reports in the literature on the addition of DMDC in passion fruit juice to preserve a fresh-like quality during low-temperature storage. Therefore, this work investigates the efficacy of DMDC in comparison with conventional pasteurization on the spoilage microbes and pathogenic microbial growth inhibition kinetics, physicochemical quality, bioactive characteristics, and shelf life of passion fruit juice at a temperature of 4 °C.

## 2. Materials and Methods

### 2.1. Passion Fruit Juice Procurement and Preparation

Freshly harvested purple passion fruit were brought from the ‘Research Institute for the Development of Natural Resources and the Environment in Chiang Mai, Thailand’. Passion fruit samples without any bruises and of uniform size were decontaminated for 2 min in a 200 ppm of sodium hypochlorite solution [16]. After the washing process, the fruits were dissected into halves to extract a yellowish fruit pulp. A passion-fruit-pulp-to water-ratio of 1:1 (*v*/*v*) was used in the preparation of passion fruit juice (PFJ), and seeds were filtered from the juice using a sterile muslin cloth. PFJ was kept at −18 ± 1 °C for further processing and analysis.

### 2.2. Pathogenic Bacteria and Microbial Growth Inhibition Kinetic Model

The pathogenic bacterial strain of *Escherichia coli* TISTR 117 was procured from the Thailand Institute of Scientific and Technological Research in Pathum Thani, Thailand [17]. *E. coli* TISTR 117 was grown in tryptic soy broth (TSB; Difco, Detroit, MI, USA) at 37 °C for 24 h. PFJ was divided into two portions: the first one was the *E. coli*-inoculated portion and the second portion was without *E. coli*-inoculation. PFJ samples without and with inoculation were incubated for 24 h at 25 ± 2 °C to attain 10^6^ CFU/mL of *E. coli*, the total viable microbes and the yeast and mold population, respectively. Furthermore, dimethyl dicarbonate (DMDC) at different doses of 50, 100, 150, 200 and 250 ppm were added to the PFJ samples, respectively. PFJ without the addition of DMDC served as a control.

### 2.3. Microbial Inhibition Growth Kinetics in PFJ

PFJ samples were assessed for microbial growth inhibition using zero- and first-order inhibition rate kinetics after the addition of varying amounts of DMDC [12]. In brief, the samples were diluted serially using a 0.85% NaCl (*w*/*v*) saline buffer. The total viable count and the count of *E. coli* were determined using the pour plate technique, while the spread plate technique was used to determine the count of yeast and mold. Furthermore, the following types of agar were utilized to culture the samples: eosin methylene blue (Difco, USA) for *E. coli*, potato dextrose agar (Himedia, Mumbai, India) for the yeast and mold count, and plate count agar (Himedia, India) for the total viable count. The incubation temperature for *E. coli* and the total viable count was 37 °C for 48 h, whereas the yeast and mold count was obtained up to 72 h at 25 °C. The microbial load was expressed as average values of log CFU/mL.

The inhibition kinetics were studied in PFJ samples with 50–250 ppm of DMDC added after 2 h of incubation. The residual microbial population and DMDC concentration were graphically presented as plots for zero-order and first-order kinetics [18]. The kinetics of microbial inhibition by DMDC were investigated by analyzing the residual live microbial populations and corresponding DMDC concentrations using two different mathematical models: zero-order kinetic (Equation (1)) and first-order kinetic (Equation (2)). The equations are as follows:

Zero-order kinetic N = N_0_ − k_0_c (1)

First-order kinetic ln[N] = ln[N_0_] − k_1_c (2)

where “N_0_” is the initial microbial content (CFU/mL), “N” is the number of residual live microorganisms (CFU/mL), “k_0_” is the zero-order inhibition rate constant, “k_1_” is the first-order inhibition rate constant and “c” is the DMDC concentration (ppm).

### 2.4. Analysis of Physicochemical Properties of PFJ Treated with DMDC

The pH of PFJ samples treated with DMDC (0–250 ppm) and the control (without any treatment) was determined using a calibrated pH meter (Mettler Toledo, S220, Greifensee, Switzerland). The total soluble solid in the range of 0–30 °Brix was determined using a digital hand refractometer (Hanna Instrument, 96801, Woonsocket, RI, USA) [13]. To obtain the ∆E value of the PFJ samples, the following formula was used (Equation (3)):

∆E = [(L*x − L*y) ^2^ + (a*x − a*y)^2^ + (b*x − b*y)^2^]1/2 (3)

In this Formula (3), the letters ‘x’ and ‘y’ indicate the initial and final color values of the PFJ samples, and the variables L*, a*, and b* represent the different dimensions of color space. These color characteristics were measured using a chroma meter (Minolta, Model CR-300 series, Osaka, Japan) [19]. The total phenolic compound (TPC), total flavonoid content (TFC), and antioxidant activity using DPPH and FRAP assays were determined following the previously described methods [20]. The analysis of ascorbic acid (vitamin C) content was conducted using the titration method. The standard solutions of 0.1% ascorbic acid and 0.1% dichlorophenol indophenol were used, and the ascorbic acid content was expressed as mg/100 mL sample [18] and calculated using the following equation:

Ascorbic acid (vitamin C) content = {Volume 0.1%ascorbic acid solution (mL)/Amount of pomegranate juice (mL) × 100}

### 2.5. Physicochemical and Microbial Analysis of DMDC-Treated, and Pasteurized PFJ during Storage at 4 °C

PFJ without DMDC served as a control during refrigerated storage. The PFJ sample with 250 pm of DMDC was compared with the conventional method of pasteurization. The PFJ samples of 300 mL volume were transferred in glass bottles before being sealed with a lug-capping machine (Shanghai Harvest Electronics Co., Ltd. Shanghai, China), subjected to pasteurization at 72 °C for 15 s, and immediately cooled in an ice bath. A digital thermocouple (Digi-Sense, Cole Parmer Instrument Co., Veron Hills, IL, USA) was used to monitor the temperature of the central cold point of the PFJ bottles throughout the pasteurization process. The microbial and physicochemical quality parameters of all PFJ samples, including the control sample (without DMDC), the DMDC-treated sample (250 ppm), and the pasteurized sample, were evaluated every 3 days for 27 days using the methods previously described in Section 2.3 and Section 2.4.

### 2.6. Statistical Analysis

All the samples were analyzed in triplicates (*n* = 3). An analysis of variance (ANOVA) of the data was conducted using completely randomized design (CRD) (SPSS version 22). The mean values were compared using Tukey’s honestly significant difference test at a confidence level of 95%.

## 3. Results and Discussion

### 3.1. Efficacy of DMDC on Microbial Inhibition in PFJ

**Figure 1:**
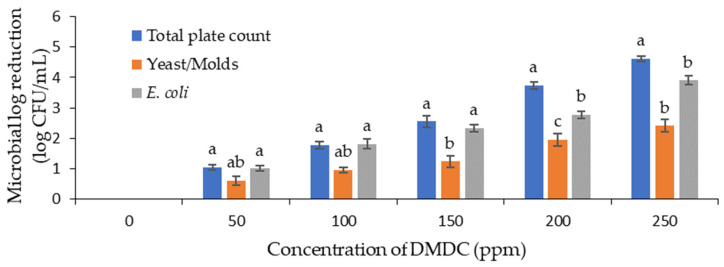
Logarithmic reduction in total viable count (TVC), yeast and mold count and *Escherichia coli* (*E. coli*) in PFJ with different concentrations of DMDC added. Values are presented as mean ± standard deviation (*n* = 3). Different lowercase letters (a–c) on the bars indicate a significant difference (*p* ≤ 0.05) within the same DMDC concentration. PFJ: passion fruit juice; DMDC: dimethyl decarbonate.

The initial count of the total viable microorganisms, yeast and mold, and *E. coli* in the PFJ sample was 5.70, 5.03, and 5.39 log CFU/mL, respectively. Upon the addition of DMDC up to a concentration of 250 ppm, the microbial counts were significantly reduced for the total viable count, yeast and mold count, and *E. coli* count (Figure 1). At a DMDC concentration of 250 ppm, the log reduction for the total viable count, yeast and mold count, and *E. coli* count was approximately 2.10, 3.35, and 3.66 log CFU/mL, respectively. In a similar study conducted with mandarin orange juice, a 5 log CFU/mL reduction in *E. coli* O157:H7 was achieved using a 250 ppm DMDC concentration [11].

**Figure 2:**
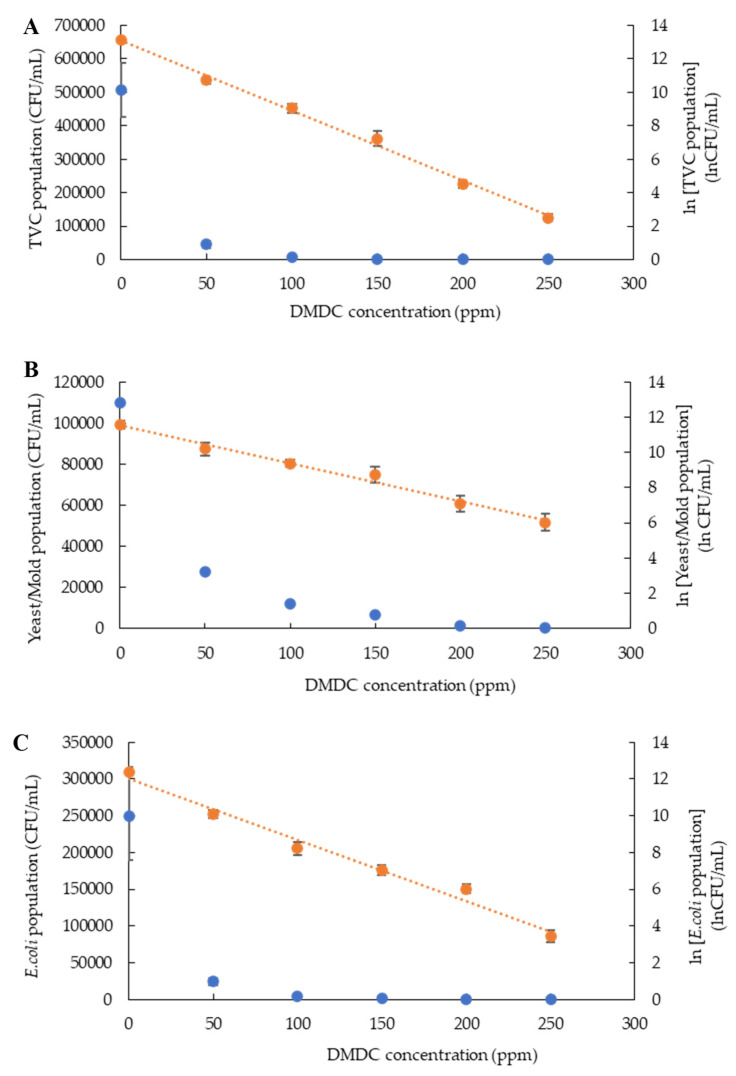
Kinetic inhibition population of total viable count (**A**), yeast and mold count (**B**) and *Escherichia coli* (**C**) in PFJ with different concentrations of DMDC added and analyzed via zero-order (blue color) and first-order (orange color) kinetic inhibition models. Values are presented as mean ± standard deviation (*n* = 3). TVC: total viable count; DMDC: dimethyl dicarbonate.

Kinetic growth inhibition model studies critically monitor the safety and predict changes in the microbial population of food that affect quality and consumer acceptance [21]. The zero- and first-order kinetic inhibition models are potential tools to study microbial growth inhibitors in fruit juice [22]. The relationship between DMDC concentrations and the population of microorganisms (total viable count, yeast and mold count, and *E. coli*) was analyzed using both zero-order and first-order kinetic models (Figure 2A–C).

The graphs plotted between DMDC concentration and the microbial population showed that the first-order kinetic model, represented by ln values of the microbial count, had a higher coefficient of determination (*R*^2^: 0.98–0.99) compared to the zero-order kinetic model coefficient (*R*^2^: 0.50–0.64) (Table 1). This indicated that the inhibition of microorganisms by DMDC followed the first-order kinetic model. Consistently, Jafari, Pongsarn, Srestasupana, Wetchasart and Assatarakul [13] observed that the reduction in *E. coli*, yeast and mold, and the total viable count followed the first-order kinetic model, with a coefficient of determination (*R*^2^: 0.93–0.96). The rate constant (k) of the kinetic model explains the rate at which the microbes decline with increasing concentrations of DMDC. On the other hand, if the reaction constant is small, it implies that DMDC concentrations slightly affect the number of microorganisms. From this perspective, k-values can be used to explain the effectiveness of DMDC in inhibiting the growth of individual microorganisms. In the current study, the highest k-value was observed in the total microorganisms (0.0420), demonstrating that DMDC could efficiently reduce the total number of microorganisms (Table 1).

Microbial growth degrades the quality of fruit juices by affecting essential nutrients and bioactive compounds. It affects changes in food quality and consumer safety. This can lead to consumer disapproval and can lead to an epidemic of foodborne diseases [11], so it is necessary to control the production process hygienically. To eliminate the growth of microorganisms in fruit juices or processed fruit products and prevent microbial contamination during the production process to bring it to a safer level, beverage industries still rely on conventional heat pasteurization. However, heat causes the loss of some important nutrients such as ascorbic acid and antioxidant activities [23]. This necessitates the possible addition of food additives generally recognized as safe (GRAS) with potential antimicrobial properties as an alternative for entrepreneurs in general to safeguard the product quality without economic losses. DMDC inhibited the growth of pathogenic and spoilage microorganisms in passion fruit smoothies and pomegranate juice subjected to different doses [12].

### 3.2. Impact of DMDC on the Antioxidant and Physicochemical Quality Changes in PFJ

Fruits and vegetables contain phenolic compounds and flavonoids with antioxidant activities which are important in balancing redox reactions to prevent diseases in the human body [24,25]. TPC and TFC ranged from 642.86 to 647.62 mg GAE/L and 887.14 to 882.18 mg QE/L, respectively, using different concentrations of DMDC (0–250 ppm) in passion fruit juice (*p* > 0.05), as shown in Table 2. Similar results of DMDC treatment at various doses on TPC and TFC contents with insignificant differences were reported in coconut water [18]. The study analyzed the antioxidant properties of phytochemicals in pomegranate and mango/passion fruit smoothies treated with different doses of DMDC using DPPH and FRAP assays. The results showed no significant differences (*p* > 0.05) in comparison to the untreated control, indicating that the addition of DMDC did not affect the antioxidant quality of the juices. These findings were consistent with the results of a shelf-life study of DMDC-fortified pomegranate juice and mixed mango/passion fruit smoothies during cold storage, which also showed no negative effects on antioxidant properties [13].

Ascorbic acid is an exogenous redox form of vitamin C, required to be supplemented in diets for human consumption to balance the redox reaction mechanism that could prevent chronic ailments such as cancer and cardiovascular diseases [26]. In addition, ascorbic acid is used as an index for quality measurement during production and food storage processes, since ascorbic acid is prone to oxidation via light, pH, radiation and temperature [27]. DMDC at various concentrations showed no negative impact on ascorbic content, ranging from 48.51 to 50.99 mg/100 mL in PFJ samples (*p* > 0.05), as shown in Table 2. In line with our results, Chen et al. [28] reported that no effect was observed on ascorbic acid content in Chinese cabbage after the addition of DMDC in dipping solution.

One of the key factors that affects fruit juice quality is pH which affects microbial growth. However, microorganisms can use fruit sugars and nutrients in juices, resulting in a decreased total amount of soluble solid. The decline in total soluble solid (°Brix) could be due to microbial metabolic activities that convert sugars into organic acids, resulting in a pH drop, and spoiled fruit juice with a short shelf-life [29]. The pH and total soluble solid content were in the range of 3.00–3.01 and 8.13–8.25 °Brix, respectively (*p* > 0.05), as indicated in Table 2. Total soluble solid (°Brix) values of 11.2, 12.5, and 10.3 for passion fruit, pineapple, and mango pulps, respectively, reported by De Farias Silva et al. [30], were higher than the values for PFJ treated with different doses of DMDC. This variation in total soluble solid was correlated with the harvesting season and level of fruit ripeness in the case of PFJ. Yu et al. [31] who studied the impacts of high-pressure homogenization and DMDC on physicochemical properties of mulberry juice and reported no marked differences in terms of pH and total soluble solid at 250 ppm of DMDC.

The color properties of PFJ samples with different DMDC concentration added presented different ranges of L* values from 52.01 to 52.50, a* values from −1.52 to −1.38 and b* values from 39.18 to 39.83 (*p* > 0.05), as displayed in Table 2. The color properties of the PFJ samples were not affected in comparison with the control (without DMDC). The color of the fruits and vegetables with diverse variations are basically the natural pigments composed of flavonoids such as anthocyanins + anthoxantins (red, blue, purple), chlorophyll (green), and carotenoids (yellow, red, orange) that ensures consumer acceptance [32]. Carotenoids, anthocyanins, and betalains are the most commonly used natural food pigments to alleviate the risk of certain chronic diseases such as type 1 diabetes, obesity, and coronary diseases [33]. The fading of color in pigmented fruit juices could be due to the degradation of color compounds via thermal treatment, enzymes or redox reactions. Thermal stabilities evaluated at 80, 95 and 110 °C of lycopene and β-carotene in tomato pulp and pink grapefruit juice showed major changes in pigment degradation [34]. Similarly, emerging technologies efficiently prevented color changes in fruits, vegetables and fruit juices [35]. DMDC addition had no impact on the color values of fermented litchi juice for 4 weeks, compared to thermal treatment during storage at 4 °C [36].

### 3.3. Effect of DMDC (250 ppm) vs. Pasteurization on Microbial Populations in PFJ during Storage at 4 °C

**Figure 3:**
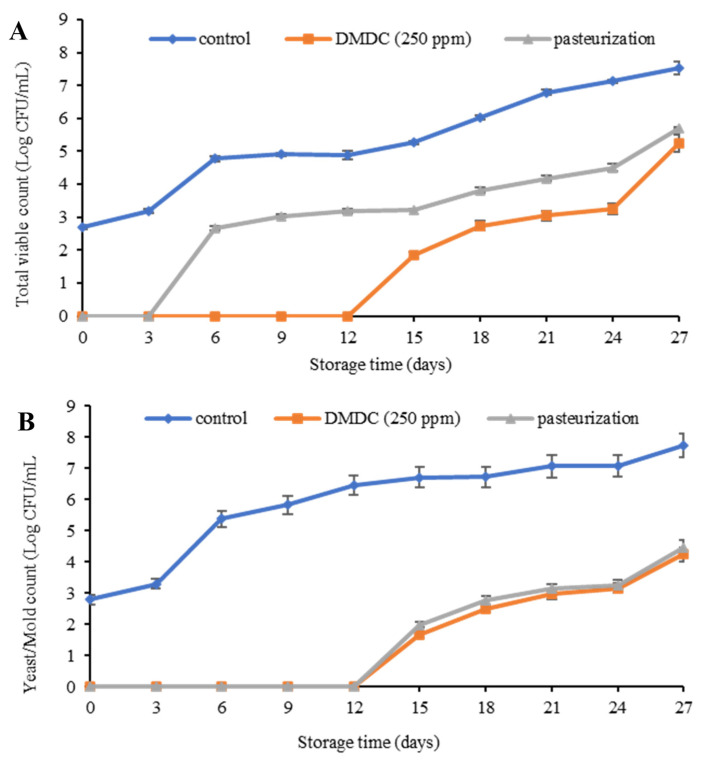
Total viable count (**A**) and yeast and mold count (**B**) of control, DMDC-added and pasteurized passion fruit juice samples during storage at 4 °C. Values are presented as mean ± standard deviation (*n* = 3). Control: passion fruit juice (PFJ) without any treatment; DMDC (250 ppm): PFJ treated with 250 ppm of dimethyl dicarbonate; pasteurization: PFJ pasteurized at 72 °C for 15 s (without DMDC).

Total viable count of spoilage microbes analyzed in PFJ samples subjected to pasteurization and DMDC treatment is presented in Figure 3A. The control sample (without any treatment) showed an initial microbial count of 2.70 log CFU/mL at the first day of storage compared to the pasteurized and DMDC treated PFJ samples (*p* ≤ 0.05). During 0–12 days, no microbial growth was found in the PFJ sample with DMDC (250 ppm) compared to untreated control (*p* ≤ 0.05), while the pasteurized sample showed microbial growth at the beginning of day 3 of storage. In general, all the samples revealed increases in total live microbes throughout 27 days of refrigerated storage. However, on day 15 of refrigerated storage, the untreated control sample exceeded the total viable count up to 5.26 log CFU/mL, while the values for PFJ with DMDC (250 ppm) and pasteurized PFJ samples were 1.86 and 3.23 log CFU/ mL, respectively. The lowest total viable count of microbes attained in PFJ treated with 250 ppm of DMDC was attributed to the strong antimicrobial power of DMDC to hinder the growth of microbes during the entire storage. A study conducted on the efficacy of DMDC added in a 40% lemon carbonated soft drink (LCSD) and 70% noncarbonated orange juice reported that the lowest microbial count was obtained in the LCSD sample [15]. DMDC (250 ppm) and nisin (200 IU/L) synergistically eliminated *Bacillus* sp. and *Leuconostoc mesenteroides* in the litchi juice [37].

The total yeast and mold count analyzed in PFJ samples subjected to pasteurization and DMDC treatment is shown in Figure 3B. The growth of yeast and mold was not found in PFJ with added DMDC and the pasteurized samples until 12 days of storage, unlike the untreated PFJ sample. The control sample without any treatment showed an augmented growth in the yeast and mold count at the very beginning of storage. With the progression of storage time, the yeast and mold count was also found to increase in all samples during the entire storage period of 27 days. Additionally, DMDC and the pasteurized samples followed a similar trend of increment from day 12 to 27 days of storage. It was observed that both DMDC and pasteurized PFJ samples exceeded the benchmark level of acceptability at 4 log CFU/mL on day 27 of storage. Based on the yeast and mold screening of PFJ, the shelf-life of the untreated control was estimated up to 3 days, and PFJ with DMDC (250 ppm) and the pasteurized samples for about 24 days. Yeast and mold are the main causes of juice deterioration as the growth of yeast and mold accelerates the fermentation process, which in turn deteriorates sensorial qualities such as appearance and taste, and so it becomes important to control the amount of yeast and mold up to 4 log CFU/mL [38]. The fungal spoilage of orange juice with 200 ppm of DMDC at refrigerated storage resulted in a 4 log reduction in yeasts and mold [38].

### 3.4. Impact of DMDC (250 ppm) and Thermal Pasteurization on Physicochemical and Antioxidant Properties of PFJ during Storage at 4 °C

**Figure 4:**
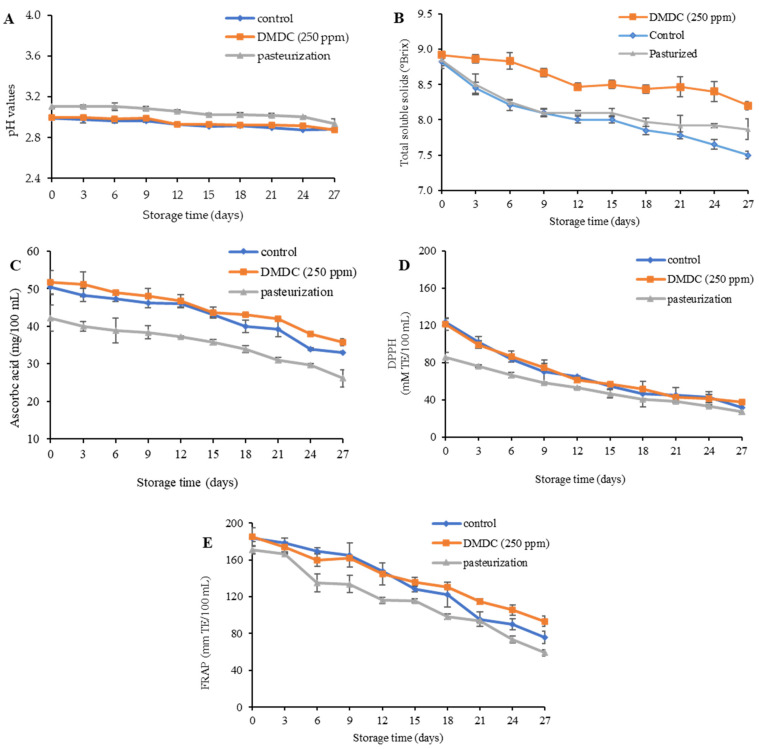
pH values (**A**), total soluble solid (**B**), ascorbic acid (**C**), antioxidant activity via DPPH (**D**) and FRAP assay (**E**) of DMDC-added and pasteurized passion fruit juice samples during storage at 4 °C. Values are presented as mean ± standard deviation (*n* = 3). Control: passion fruit juice (PFJ) without any treatment; DMDC (250 ppm): PFJ treated with 250 ppm of dimethyl dicarbonate; pasteurization: PFJ pasteurized at 72 °C for 15 s (without DMDC).

The pH of the untreated control, 250 ppm DMDC and pasteurized PFJ samples was in the range of 2.93–3.16 at day 0 of storage, as shown in Figure 4A. The pasteurized sample showed a slightly elevated trend in pH compared to the other samples, possibly due to thermal changes induced in the PFJ constituents at the beginning of storage study. Thereafter, the pH of all samples showed a decreasing trend during 27 days of refrigerated storage. This declining trend was in line with Lan et al. [39] who studied the quality and shelf-life of fresh mango juice, reporting that microorganisms could degrade sugars in juice as an energy source and produce organic acids as a byproduct of carbohydrates through metabolic pathways. The total soluble solid (TSS) represents the amount of sugar in juice, which is an important factor for taste and sensory properties. Figure 4B presents the total amount of TSS of the PFJ samples without and with treatments. According to the results, from day 0 to 27 days of storage, the TSS in the control, 250 ppm DMDC and pasteurized samples decreased from 8.82 to 7.50, 8.92 to 8.20, and 8.85 to 7.87 °Brix, respectively. The lowest decreases in TSS were visualized in the 250 ppm DMDC sample compared to the control and pasteurized PFJ samples. Additionally, no difference was observed in the untreated control and pasteurized PFJ samples up to 18 days of refrigerated storage. After 18 days of storage, the TSS of the untreated control showed a marked decrease in comparison with the pasteurized and DMDC-treated samples until the end of storage (day 27).

Vitamin C, also termed ascorbic acid, is inherent in fruits and marks an essential nutrient for the well-functioning of enzymes and the immune system of the human body. Many juice products with high levels of ascorbic acid are therefore popular among consumers, and the decrease in the amount of ascorbic acid in fruits indicates the quality degradation of juice. It was found that when the storage time was augmented, the ascorbic acid content was more likely decreased, as shown in Figure 4C. The initial ascorbic acid content of 50.36, 51.68 and 42.20 mg/100 mL at day 0 of storage was measured in the control, 250 ppm DMDC and pasteurized PFJ samples, respectively. The ascorbic acid values of the control, DMDC-treated and pasteurized PFJ samples were 33.08, 35.72 and 26.11 mg/100 mL, respectively, at day 27 of storage. As predicted, the pasteurized sample had a smaller amount of ascorbic acid which is sensitive to heat. It can be easily degraded by ascorbic acid oxidase. Pulsed electric field treatment (30 kV/cm and 100μs) preserved 50% of ascorbic acid losses in 277 days, in comparison with the pasteurized orange juice that could safeguard ascorbic acid until 90 days of storage at 2 °C [40].

The DPPH and FRAP assays employed for the assessment of antioxidant activity tended to decrease during cold storage (Figure 4D,E). The initial DPPH values in the control, DMDC-treated and pasteurized PFJ samples were 124.11, 121.61 and 85.78 mM TE/100 mL, respectively. At the end of the storage period, the antioxidant activity measured via the DPPH method was reduced to 31.54, 37.54 and 27.27 mM TE/100 mL in the control, DMDC-treated and pasteurized PFJ samples (Figure 4D). The FRAP values of the control, 250 ppm DMDC and pasteurized samples were 183.66, 185.32 and 170.69 mM TE/100 mL, respectively. The FRAP values also decreased to 75.88, 93.1 and 59.03 mM TE/100 mL at the end of refrigerated storage (day 27). Despite this fact, marked differences were attained in the DPPH and FRAP values of the pasteurized sample in comparison with the 250 ppm DMDC sample during intervals of 27 days of storage. In line with our results, Porto et al. [41] reported that the antioxidant activity of beetroot juice mixed with orange juice during storage at 4 °C for 30 days decreased. Mgaya-Kilima et al. [42] also showed a decreasing trend in the antioxidant activity of okra juices mixed with other juices (mango, papaya and guava) stored at 4 °C and 28 °C for 6 months.

**Figure 5:**
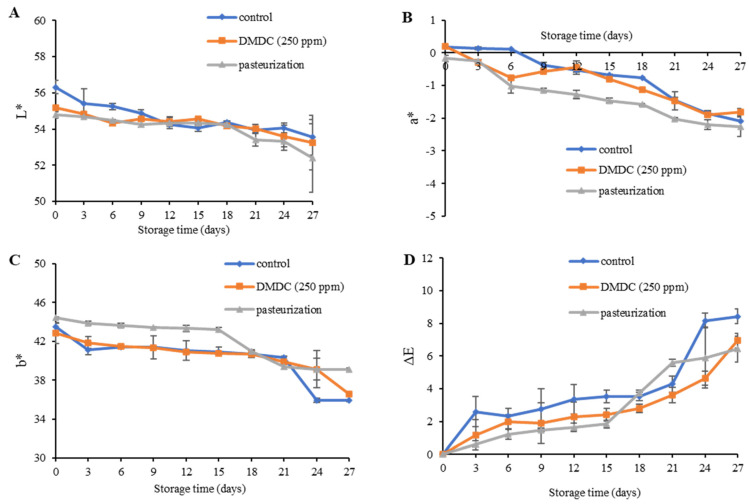
L* values (**A**), a* values (**B**), b* values (**C**) and ΔE values (**D**) of DMDC-added and pasteurized passion fruit juice samples during storage at 4 °C. Values are presented as mean ± standard deviation (*n* = 3). Control: passion fruit juice (PFJ) without any treatment; DMDC (250 ppm): PFJ treated with 250 ppm of dimethyl dicarbonate; pasteurization: PFJ pasteurized at 72 °C for 15 s (without DMDC); ΔE values represent the color difference in PFJ.

The color changes are signs of food deterioration and are possibly due to enzymatic browning and the release of hormones responsible for overripening hormones [43]. Changes in the appearance of food products affect consumer acceptance and decrease the commodity value. During the cold storage for 27 days, the color values (L*, a* and b*) decreased. The L* value decreased from 56.32 to 52.40, the a* value was in the range of −2.27 to −0.20, and the b* value ranged from 35.89 to 44.44. A decrease in L*, a* and b* leads to incremental changes in the ∆E value of all samples during the entire storage (Figure 5A–D). Correspondingly, Hu et al. [44] who studied the quality and shelf-life of pasteurized jabuticaba grape juice during storage at 4 °C for 28 days reported that the ∆E tended to increase throughout the storage period. However, the DMDC had no impact on the color attributes of PFJ. In fact, some changes might have occurred during the thermal pasteurization of the PFJ sample as opposed to the untreated control sample. The L* value of heat-treated litchi juice showed a decreased trend, possibly due to the aggregation of protein and pulp fiber, and the b* value slightly increased due to the caramelization of heated sugars [36].

## 4. Conclusions

DMDC at 250 ppm exhibited significant reductions in *E. coli*, yeast and mold count, as well as the total viable count (TVC) in PFJ samples, compared to the untreated controls. Despite this, no changes were observed in physical, chemical, or antioxidant properties measured via DPPH and FRAP assays. Over 24 days of refrigerated storage, DMDC 250 ppm showed a 54.35% reduction in TVC and a 53.95% reduction in yeast and mold populations compared to the untreated samples. The pH, TSS, ascorbic acid content, and antioxidant activity remained unaffected by DMDC treatment. Minimal changes in color values were observed, indicating the preservation of product quality. Overall, DMDC at 250 ppm presents a cost-effective preservation method for juice manufacturing, ensuring both quality retention and safety for consumers.

## Figures and Tables

**Table 1:** Effects of DMDC on the kinetic growth inhibition of spoilage and pathogenic microbes in passion fruit juice.

Microorganism	Zero-Order Model		First-Order Model	
	Rate Constant (k_0_)	Coefficient of Determination (*R*^2^)	Rate Constant (k_1_)	Coefficient of Determination (*R*^2^)
Total viable count	1529.80	0.50	0.04	0.99
Yeast and mold	361.52	0.64	0.03	0.98
*E. coli*	756.73	0.50	0.03	0.98

k_0_ = zero-order kinetic inhibition rate constant. k_1_ = first-order kinetic inhibition rate constant. DMDC: dimethyl dicarbonate.

**Table 2:** Screening of chemical, color and antioxidant properties of passion fruit juice subjected to different levels of DMDC at 2 h of refrigerated storage.

Sample Analysis	DMDC Concentration (ppm)					
	0	50	100	150	200	250
pH ^ns^	3.00 ± 0.01	3.00 ± 0.03	3.00 ± 0 01	3.01 ± 0.01	3.00 ± 0.01	3.00 ± 0.32
°Brix ^ns^	8.13 ± 0.18	8.25 ± 0.35	8.13 ± 0 18	8.25 ± 0.35	8.25 ± 0.35	8.13 ± 0.18
Color values						
L* ^ns^	52.07 ± 0.21	52.01 ± 0.18	52.01 ± 0.19	52.22 ± 0.15	52.30 ± 0.21	52.50 ± 0.01
a* ^ns^	−1.44 ± 0.04	−1.38 ± 0.06	−1.39 ± 0.08	−1.52 ± 0.04	−1.43 ± 0.06	−1.46 ± 0.01
b* ^ns^	39.18 ± 0.62	39.39 ± 0.18	39.41 ± 0.12	39.30 ± 0.23	39.66 ± 0.01	39.83 ± 0.11
Ascorbic acid ^ns^	50.32 ± 0.01	49.19 ± 0.01	50.99 ± 0.09	49.86 ± 0.06	49.41 ± 0.35	48.51 ± 0.60
TPC ^ns^	645.79 ± 0.20	644.32 ± 0.40	647.62 ± 0.01	644.69 ± 0.56	644.69 ± 0.84	642.86 ± 0.91
TFC ^ns^	882.18 ± 0.05	879.38 ± 0.20	877.14 ± 0.43	880.50 ± 0.76	880.50 ± 0.83	879.94 ± 0.11
DPPH ^ns^	108.83 ± 0.24	114.67 ± 0.21	108 ± 0.16	107.17 ± 0.34	107.17 ± 0.39	105.92 ± 0.54
FRAP ^ns^	180.28 ± 0.37	181.11 ± 0.29	180 ± 0.18	177.78 ± 0.56	180.56 ± 0.09	178.33 ± 0.69

The values in the table are presented as the mean ± standard deviation, and superscript ^ns^ denotes not significantly different (*p* > 0.05) within same row. TPC: total phenolic content (mg GAE/ L); TFC: total flavonoid content (mg QE/ L); ascorbic acid (mg/100 mL); DPPH: 2,2-diphenyl-1-picrylhydrazyl (mM TE/100 mL); and FRAP: ferric reducing antioxidant power (mM TE/100 mL). DMDC: dimethyl dicarbonate. Color properties are indicated by L*, a* and b* values and are commonly measured such that L* is 0, implying that the sample is black, and if L* is 100, the sample is white, while a* value denotes red and green, and positive means that the color value tends towards red and vice versa, and b* value designates yellow (+) and blue (−).

## Data Availability

The original contributions presented in the study are included in the article, further inquiries can be directed to the corresponding authors.
